# Genome-Wide Association Studies of HIV-1 Host Control in Ethnically Diverse Chinese Populations

**DOI:** 10.1038/srep10879

**Published:** 2015-06-03

**Authors:** Zejun Wei, Yang Liu, Heng Xu, Kun Tang, Hao Wu, Lin Lu, Zhe Wang, Zhengjie Chen, Junjie Xu, Yufei Zhu, Landian Hu, Hong Shang, Guoping Zhao, Xiangyin Kong

**Affiliations:** 1 The Key Laboratory of Stem Cell Biology, Institute of Health Sciences, Shanghai Institutes for Biological Sciences, Chinese Academy of Sciences, and Shanghai Jiao Tong University School of Medicine, Shanghai 200031, People’s Republic of China; 2 Department of Laboratory Medicine, National Key Laboratory of Biotherapy/Collaborative Innovation Center of Biotherapy, and Cancer Center, West China Hospital, Sichuan University, Chengdu, China; 3 CAS-MPG Partner Institute and Key Laboratory for Computational Biology, Shanghai Institutes for Biological Sciences, Chinese Academy of Sciences, Yueyang Road 320, Shanghai 200031, People’s Republic of China; 4 Infectious Diseases Department, Beijing You’an Hospital, Capital Medical University, Beijing 100069, People’s Republic of China; 5 Yunnan Provincial Center for Disease Control and Prevention, Kunming 650022, People’s Republic of China; 6 Henan Provincial Center for Disease Control and Prevention, Zhengzhou 450016, People’s Republic of China; 7 Yili Prefecture Center for Disease Control and Prevention, Yining 835000, Xinjiang, People’s Republic of China; 8 Key Laboratory of AIDS Immunology of National Health and Family Planning Commission, Department of Laboratory Medicine, The First Affiliated Hospital, China Medical University, Shenyang 110001, People’s Republic of China; Collaborative Innovation Center for Diagnosis and Treatment of Infectious Diseases, Hangzhou 310000, People’s Republic of China; 9 Shanghai-MOST Laboratory of Disease and Health Genomics, Chinese National Human Genome Center at Shanghai, Shanghai 201203, People’s Republic of China

## Abstract

Genome-wide association studies (GWASs) have revealed several genetic loci associated with HIV-1 outcome following infection (e.g., *HLA-C* at 6p21.33) in multi-ethnic populations with genetic heterogeneity and racial/ethnic differences among Caucasians, African-Americans, and Hispanics. To systematically investigate the inherited predisposition to modulate HIV-1 infection in Chinese populations, we performed GWASs in three ethnically diverse HIV-infected patients groups (i.e., HAN, YUN, and XIN, N = 538). The reported loci at 6p21.33 was validated in HAN (e.g., rs9264942, *P* = 0.0018). An independent association signal (rs2442719, *P* = 7.85 × 10^−7^, HAN group) in the same region was observed. Imputation results suggest that haplotype HLA-B*13:02/C*06:02, which can partially account for the GWAS signal, is associated with lower viral load in Han Chinese. Moreover, several novel loci were identified using GWAS approach including the top association signals at 6q13 (*KCNQ5*, rs947612, *P* = 2.15 × 10^−6^), 6p24.1 (*PHACTR1*, rs202072, *P* = 3.8 × 10^−6^), and 11q12.3 (*SCGB1D4*, rs11231017, *P* = 7.39 × 10^−7^) in HAN, YUN, and XIN groups, respectively. Our findings imply shared or specific mechanisms for host control of HIV-1 in ethnically diverse Chinese populations, which may shed new light on individualized HIV/AIDS therapy in China.

Human immunodeficiency virus infection/acquired immunodeficiency syndrome (HIV/AIDS) has been reported to be the leading cause of death among infectious diseases in China. According to Chinese official statistical reports, the total number of HIV patients reached 780,000 in 2012, with 17,740 dying within the first ten months, an increase of 8.6% compared with 2011^1^. It is well-known that HIV-1 infection exhibits considerable phenotypic heterogeneity at different levels including virus acquisition^2^, disease progression^3^, and response to treatment^4^. Accumulating evidence in recent decades indicates that inherited predispositions correlate with this phenotypic heterogeneity^5^, and these data have been supported by genome-wide association studies (GWASs), which identified several genetic loci at 6p21.33 (e.g., rs2395029, rs9264942) that influenced HIV-1 infection outcomes in Caucasians^6^,^7^. The major histocompatibility complex (MHC) genes (e.g., *HLA-B*, *HLA-C*) in this region were believed to be involved^5^. Similar GWASs were performed in African Americans populations but targeted to different SNP (i.e., rs2523608) and *HLA* allele^8^,^9^, indicating racial/ethnic disparities in the host control of HIV-1. Therefore, it is important to perform GWAS in other ethnicities, such as Chinese populations, to identify common and ethnicity-specific association loci.

The classical class I human leukocyte antigens HLA-A, HLA-B and HLA-C are capable of presenting a wide variety of antigens, such as viral peptides, to alert antigen-specific CD8 + T cells and nature killer cells during immune responses, which depends upon the high variations in HLA proteins, particularly of the extracellular peptide domain^10^. Associations of *HLA* loci with host control of HIV-1 infection outcomes indicates the important role of *HLA* class I alleles on the immune response of cytotoxic T lymphocytes (CTLs) for host control of HIV-1^11^,^12^,^13^,^14^,^15^. Furthermore, the international HIV controllers study implicated specific *HLA* alleles including HLA-B*57:01, HLA-B*27:*05 and HLA-C* as the most popular candidates for variance of HIV-1 set point in steady state in Europeans, based on suggestive functional amino acids at position 97, 67 in binding groove of HLA-B and position 304 of HLA-C^7^. However, some reported association signals (e.g., rs2395029, HLA-B*57:01) are very rare in Chinese populations with minor allele frequencies (MAFs) of less than 1%, much lower than in Caucasian or African populations^16^,^17^.

Thus far, no systematic investigations of inherited predisposition to HIV-1 host control have been performed in Chinese populations. In this study, we, for the first time, performed a GWAS study in multi-ethnic Chinese patients and systematically investigated association loci and the functional genes that participate in host control of HIV-1 infection outcomes.

## Results

To comprehensively examine germline variants that were associated with host control of HIV-1 infection outcome in Chinese populations, three geographically different multi-ethnic patient groups were recruited in China including HAN, YUN, and XIN groups (Figure S1). Following QC procedures (see Methods), 538 patients remained for subsequent analyses (Table 1). HIV-1 viral load, one of the most important indicators of HIV-1 infection and progress, was estimated separately in each group and used to represent the HIV-1 infection outcomes. The median of viral loads was approximately 5 × 10^4^ cp/ml for all three groups (Figure S2). Using principal component analysis (PCA) in multi-ethnic populations (i.e., Chinese HIV patients and HapMap individuals), the top-ranked two principal components (PC1 and PC2) separated all individuals into 4 groups. HAN and YUN individuals overlapped with CHB and JPT from the HapMap projects but separated from XIN, CEU, and YRI (Figure S3). Further PCA analyses of our patients only showed that each patient group tended to cluster, indicating the strong population stratification effect. To further exclude the effects of a possible patient selection bias, the presenting features (i.e., gender, age, and transmission route of HIV infection) were considered as covariates. Finally, we preformed GWASs on the phenotype of HIV-1 viral-load set-point in each patient group separately using linear regression and adjusting for the presenting features (gender was excluded for Yun and Xin group associations for minority women) and the top five PCs. Quantile-quantile (Q-Q) plots showed that the inflation of the genome-wide association test statistics was inappreciable in our analyses (Figure S4).

**Figure 1:**
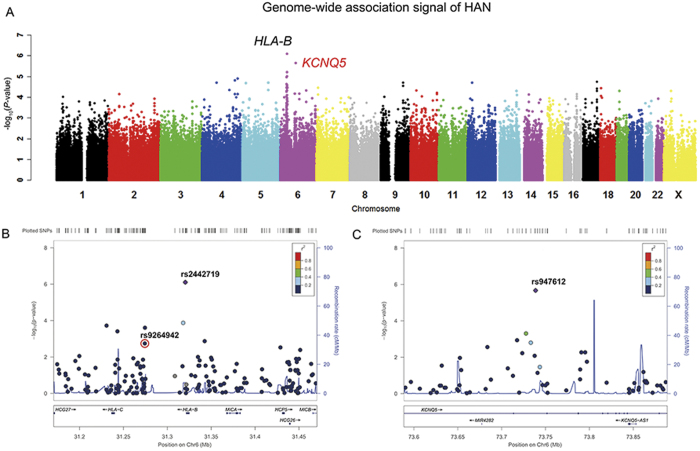
Manhattan plot for host control of HIV-1 outcome following infection in the HAN group. (**A**) Associations between genotype and HIV-1 viral-load set-point were evaluated using a linear regression model for 481,717 SNPs in the HAN group (N = 280). *P* values (-log_10_ *P*, y axis) were plotted against respective chromosomal position of each SNP (x-axis). Gene symbols are indicated for the top 2 loci, and the novel association SNP located gene *KCNQ5* is highlighted in red. (**B**) and (**C**) Regional plots showing association results for SNPs spanning Chr6: 31.15-31.5 Mb at *HLC-B* and Chr6: 73.6-73.9 Mb at *KCNQ5* locus, respectively in the HAN group. The plots were constructed using LocusZoom^46^: *P* values (-log_10_ *P*, y axis) were plotted against respective chromosomal positions of each SNP (x axis), and colors indicate LD (r^2^) with top signals in 1000 genomes from East Asian populations. The reported SNP rs9264942 (*P* = 0.0018) is highlighted within the red circle in Panel B.

**Figure 2:**
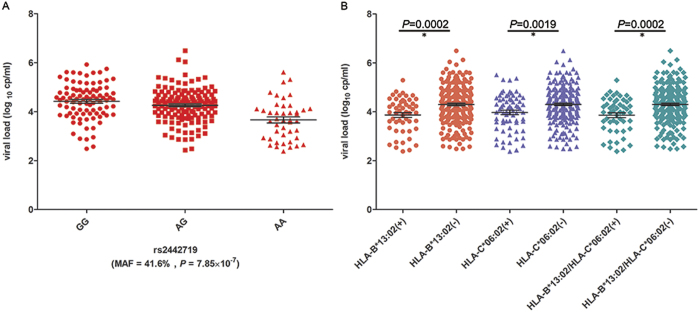
Viral-load distribution in patients with rs2442719, HLA-B*13:02 and HLA-C*06:02. Viral loadings of patients with the AA allele at rs2442719 (**A**) and HLA-B*13:02/HLA-C*06:02 (**B**) were significantly lower than in other patients. The black lines denote the mean value and the error bar represents the standard error of the mean. Log_10_(viral load) (y axis) were plotted against patients with different alleles (x axis). *P* values were calculated using unpaired t test with Welch’s correction.

We first focused on several independent loci around the major histocompatibility complex (MHC) region at 6p21.33 identified by previous GWASs in Caucasians and African Americans. Some of the top GWAS SNPs at the *HLA-C* locus (e.g., rs9264942, Fig. 1B) associated statistically with HIV-1 viral-load set-point in the HAN group (*P* = 0.0018) but not the YUN or XIN group (Table 2). In contrast, neither the reported top SNP in Caucasians at *HCP5* (rs2395029) nor the top association SNP (rs2523608) at *HLA-B* in African Americans showed any significant association in any of our Chinese patient groups (Table 2, Figure S6) likely due to their racial/ethnic specificity, which is further supported by the varied MAFs of these SNPs in different populations (e.g., rs2395029, Table 2). Interestingly, the top GWAS signal (rs2442719, *P* = 7.85 × 10^−7^, Fig. 1B), which reached marginal genome-wide significance (*P*_*significant*_ < 1.04 × 10^−7^ following Bonferroni correction, see Methods) in our HAN group, was very close to rs2523608 in *HLA-B*, the top signal in African Americans; however, it showed different LD patterns (i.e., r^2^ = 0.33 in YRI *vs.* r^2^ = 0.04 in CHB) between these two SNPs implying that the casual variant(s) may be tagged by different SNPs in multiple races/ethnicities. Indeed, 4 out of 5 top GWAS SNPs in the HAN group were located within the MHC region at 6p21.33 (Table S1). The top association SNP (i.e., rs2442719, described above) explained 9.5% of the total variation in HIV-1 set point in Han Chinese with the Adenine nucleotide as the protective allele, which was independent from the other top significant SNPs (i.e., rs12210887, rs3763312, rs2532924, rs1252824; *P*_*condition*_ = 0.0028) (Fig. 2A). However, none of the top association SNPs were validated in the XIN group, and only two SNPs (i.e., rs130065, rs3132580) were significant in the YUN group (Table S2). In contrast, we checked the entire MHC region at 6p21.33 in the YUN and XIN groups, and weak associations were observed at rs494620 (*P* = 4.3 × 10^−3^) and rs3132486 (*P* = 3.8 × 10^−3^) as the top signals in YUN and XIN (Table S3, S4) groups, respectively. These data indicated a weak impact of HLA on HIV infection outcome in these two racial/ethnic groups.

Next, we attempted to investigate the candidate HLA alleles and their polymorphic amino acids that were likely to be responsible for the host control of HIV-1 infection outcome in ethnic Han Chinese. The HLA alleles and the corresponding polymorphic amino acids were imputed using the HapMap^18^ reference panels of 89 Asian individuals who have available 4-digit class I and class II classic *HLA* genotypes^19^. Nineteen *HLA-A*, 32 *HLA-B*, 17 *HLA-C* and 50 class II *HLA* alleles were imputed and used to perform association analyses with viral-load set-points using linear regression. Two *HLA* alleles, HLA-B*13:02 and HLA-C*06:02, were identified to be significantly associated with lower viral load (*P* < 2.5 × 10^−4^ following Bonferroni correction; Table 3 and Fig. 2B) and showed strong LD (D’ = 0.979, r^2^ = 0.678) between them. Conditional tests indicated that the association of HLA-B*13:02 was independent of rs2442719, whereas HLA-C*06:02 was not (Table 3), and a moderate LD (D’ = 1, r^2^ = 0.22) was noted between rs2442719 and HLA-C*06:02. Amino acid changes in HLAs were considered the causal events of HIV-1 host control^7^, and we further investigated the imputed amino acid polymorphisms for the different HLA alleles in the HAN group. We observed multiple variants that showed statistically significant associations (Table 4) with position 156 in HLA-C (4 possible amino acids, *P* = 1.10 × 10^−4^) and position 145 in HLA-B (*P* = 4.84 × 10^−5^) ranking at the top, and which were distinct from reported HLA alleles and variants in Caucasians and African Americans^7^,^20^. These data imply that different functional variants of the same genes may contribute to HIV-1 control among races/ethnicities.

Importantly, our multi-ethnic GWASs also identified several potential novel loci for HIV-1 control in Chinese populations. The top signals were located at 6q13 (*KCNQ5*, rs947612, *P* = 2.15 × 10^−6^), 6p24.1 (*PHACTR1*, rs202072, *P* = 3.8 × 10^−6^), and 11q12.3 (*SCGB1D4*, rs11231017, *P* = 7.39 × 10^−7^) in HAN, YUN, and XIN groups, respectively (Fig. 1, S5, S7). None reached genome-wide significance – most likely due to the small sample size. However, none of the novel loci cross validated in other groups (Table S5). Transethnic meta analysis using MANTRA^21^ revealed four marginally significant SNPs (log_10_ BF > 5, Table S6); however, at this significant level, the association evidence strongly depends on prior assumptions, so the association need further validation. This results indicated racial/ethnic differences in host control of HIV-1 infection outcome among multi-ethnic populations in China.

## Discussion

The majority of GWASs are performed in European populations with recent extent studies in diverse populations revealing both similarities and differences in the genetic architecture of disease susceptibility between ethnic groups^22^. Multi-ethnic GWAS are believed to increase the power to identify more association loci and evaluate racial/ethnic specificities^22^,^23^. Inherited predispositions to HIV-1 infection outcomes has been supported by several GWASs and subsequent fine-mapping approaches in different racial/ethnic populations including Caucasians, African Americans, and Hispanics^6^,^7^,^20^. The most significant SNP at the 6p21.33 locus, located upstream of *HLA-C* (e.g., rs9264942), also correlates with the HLA-C cell-surface protein expression on primary T cells in European Americans^24^. Although association signals were consistently observed in the 6p21.33 region, dramatic racial/ethnic differences were noted^25^. Thus, for the first time, we systematically studied host control of HIV-1 infection outcomes using GWAS in three multi-ethnic Chinese groups. Some reported SNPs (e.g., rs9264942) at 6p21.33 were validated in our HAN group; however, no trend was observed in either the YUN or XIN group (Table S5), most likely due to racial/ethnic specificity rather than insufficient statistical power (e.g., sample size). Some statistically significant association signals were observed in the MHC region at 6p21.33 in the YUN (e.g., rs494620, *P* = 4.3 × 10^−3^) and XIN (e.g., rs3132486, *P* = 3.8 × 10^−3^) groups but were much weaker than those of Caucasians, African Americans, and our HAN group. These racial/ethnic differences may be explained by three possibilities: 1) the same causal variant(s) were located at 6p21.33 but were tagged by different SNPs due to the varied LD patterns among different racial/ethnic groups. For example, the top association signal in the HAN group (rs2442719) was very close to the top SNP in African Americans (rs2523608), which was not validated in any of our patient groups. Interestingly, the LD between these two SNPs was moderate in Africans (r^2^ = 0.33) and weak in East Asians (r^2^ = 0.04) implying same causal variant(s) may be tagged by different SNP markers between races/ethnicities. 2) Diverse functional variants in different ethnicities were located in the same gene(s) and that affected HIV-1 infection and progression. For example, multiple independent association signals at 6p21.33 in the HAN group were observed, which is consistent with previous findings in Caucasians and African Americans, indicating that multiple causal variants may be located there. 3) Different genes were involved in different ethnicities. For example, some functional variants may be hugely different between races/ethnicities in terms of MAF. Rare or monomorphic functional variants will likely result in loss of association in some races/ethnicities (e.g., 6p21.33 in YUN and XIN group).

We imputed the *HLA* genotypes and identified the suggestive haplotype HLA-B*13:02/C*06:02 that was associated with HIV-1 viral-load set-point in our HAN group. This result is consistent with a previous study involving 143 Chinese HIV-1 donors, in which the HLA-A*30/B*13/Cw*06 haplotype was observed to be associated with lower viral loads^26^. Although HLA-B*13:02 effects were independent of rs2442719, HLA-C*06:02 showed a moderate LD with rs2442719; thus, the haplotype HLA-B*13:02/C*06:02 may partially explain the association of rs2442719 (Table 3). However, we cannot determine the allele in the HLA-B*13:02/C*06:02 haplotype that plays the dominant role in impacting viral load, and both of these alleles have been reported to be associated with the control of HIV viral load independently. HLA-B*13:02 was previously reported as a potentially associated allele in the GWASs in Caucasians^7^,^27^, and it was confirmed to contribute to a broad Gag-specific CD8 + response that was associated with the control of viremia, with similar effects noted for HLA-B*57, B*58:01 and B*27^28^,^29^,^30^,^31^. Similarly, *HLA-C* correlated with rs9264942, which is located 35 kb upstream from *HLA-C*, and they were all associated with viremia control in Caucasians^24^,^32^,^33^,^34^. It is worth noting that there is a trend for protective *HLA-C* alleles against HIV showing strong LD with *HLA-B* alleles. For example, in Caucasians, HLA-C*06:02 shows strong LD with the protective allele HLA-B*57:01^35^. Similarly, HLA-C*06:02 shows LD with the risk allele HLA-B*58:02 in Africans^36^. Therefore, further replication in larger samples and functional analysis are required.

In addition, we performed amino-acid imputation to elucidate the potential functional amino acid positions within the HLA proteins associated with viral load (Table 4). It is noted that HLA-B position 145, located in an exposed region of the α2 helix, shows two allelic variants: leucine is expressed uniquely by HLA-B*13:01/13:02, whereas other HLA-B haplotypes are characterized by arginine^37^. Thus, leucine 145 appears to be responsible for the association of HLA-B*13:02 with viral-load control. We also observed a segregation of association signals for positions shaping the F pocket that form the C-terminal anchor and affect epitopes presenting to CD8 + T cells^7^,^37^,^38^,^39^,^40^ including positions 77, 80, 81, 84, 95, 116, 123, 143 and 147, which suggests that the preference binding pocket within HLA-B. However, positions significant in Caucasians or Africa Americans, such as 97, 67, 63, 62, were not replicated in our population most likely due to the distinct associated HLA-B alleles.

Within HLA-C, the top position 156 (Table 4) shows four allelic variants, which locate to the D binding pocket and are thought to be one of the key positions that influence T-cell allorecognition^7^,^41^, though no evidence connects it with HLA-C expression level or HIV viral control. Notably, the methionine at position 304 identified in Caucasians showed significance in our population (*P* = 3.8 × 10^−3^) and correlates with HLA-C expression levels^7^. However, all positions in HLA-C were not independent of rs2442719, indicating the proxy role of rs2442719 for HLA-C.

Importantly, several potential racial/ethnic-specific loci have been identified using our GWAS approach. Based on data from the ENCODE project^42^, SNP rs947612 and rs202072 were located in strong enhancer regions; thus, they may impact the epigenetic characteristics of *KCNQ5* and *PHACTR1*, respectively. However, due to the small sample size and the complicated population structure, additional validations are required using larger sample sizes to confirm the actual ethnicity specific association of these loci with host control of HIV-1 outcome following infection.

Host genetic variants are strongly associated with inter-individual variability in both HIV life cycle and immune responses. Our study systematically examined the common variants associated with viral load in multi-ethnic Chinese populations. The validated and newly identified HIV-1 host control loci and their cellular pathways may suggest important targets for future vaccine design and disease therapy in China.

## Material and methods

### HIV-1-infected cases

HIV-1 infected patients (N = 1,556) from three geographically different areas were recruited and clinical diagnoses were performed in the Chinese Medical University (Shenyang, Liaoning Province, China) including HAN (from the central and north of China, primarily including individuals of Han Chinese ethnicity), YUN (from the southwestern China, primarily including Han, Dai, and Jingpo Chinese ethnicities), and XIN (from northwestern China, primarily including Uygur and Hui Chinese ethnicities; Figure S1). This study was approved by the Institutional Review Board of the First Hospital of China Medical University. Informed consent was obtained from all patients and all experiments were performed in accordance with relevant guidelines. All participants showed profiles of steady-state viremia and estimated infection dates were generated. HIV-1 clades B and CRF01_AE are the major subtypes of viruses in our groups. Viral-load measurements were processed as follows for quality control (QC) purposes: 1) steady state plasma HIV RNA (viral load, VL, measured in copies/ml and transformed by log10) determinations in the absence of antiretroviral therapy used in the study should have been at least 3 months after the estimated infection date; 2) for VLs measured at 3 years after infection, CD4 cell counts must have been greater than 350 cells/mm^3^ to ensure that the patients were not within the AIDS period; 3) for VLs measured during the first year after infection, VL values that were 0.25 log10 higher than the average of the subsequent VL were excluded; 4) VL outliers, those that were 0.5 log10 higher or lower than the average of other VLs, were excluded. Finally, 538 individuals (486 males, 52 females) who had at least one available VL measurement passed the QC for further analyses, and the average VL value that satisfied the above conditions was defined as the set point (Table 1).

### Genotyping and quality control

All genomic DNA samples (N = 538) were extracted from peripheral blood samples of the patients using QIAamp Blood DNA Midi Kit (Qiagen, Germany) and then genotyped with Illumina Human660W-Quad Beadschip. Call rates of all samples were greater than 99%; however, 11 genetically related patients estimated using PLINK software^43^ were removed. SNP QC procedures were performed separately in each cohorts based on the call rate (>99%), minor allele frequency (MAF > 0.01), and Hardy-Weinberg equilibrium (*P* > 0.01). 481,717, 488,154, and 528,294 SNPs remained for further analyses in the HAN, YUN and XIN groups, respectively. Thus, *P* < 1.04 × 10^−7^, 1.02 × 10^−7^, and 9.46 × 10^−8^ were considered as genome-wide significant cutoffs for Bonferroni corrections for each group.

### Statistical analyses

To examine the population stratification, principal component analysis (PCA) was performed for the multi-ethnic populations including our Chinese HIV-infected patients and HapMap individuals from descendants of Northern Europeans (CEU, N = 112), West Africans (YRI, N = 113) and East Asians (CHB, N = 84; JPT, N = 86) using EIGENSTRAT (following the steps described previously)^6^,^44^,^45^. Similar PCAs were performed in Asian populations only (CHB + JPT + HAN + YUN + XIN), our patients alone (HAN + YUN + XIN) and each patient group alone (i.e., HAN, YUN, and XIN, separately). The top two PCs (principle components; i.e., PC1 and PC2) were used to estimate the racial/ethnic differences among each group. There were 6 samples in the HAN group and 2 samples in the XIN group that were removed because they were outliers of the populations. For each group, the top five PCs nominated by Tracy Widom tests in EIGENSTRAT were included as covariates in the GWASs.

The relationship between genotypes at each SNP and HIV-1 infection outcome, which was represented by HIV viral-load set-point, was estimated using PLINK^43^ in the linear regression model with adjustments for the presenting features (i.e., gender, age, principle components and transmission route of HIV infection). *P* values for all SNPs were then used to generate the Manhattan plot and quantile-quantile (Q-Q) plot using R (version 3.1.0) statistical software. Transethnic meta analysis was performed using MANTRA software^21^.

### Imputation of *HLA* genotypes and amino acids

We performed the imputation of HAN in the major histocompatibility complex (MHC) region (between 29 and 36 Mb on Chromosome 6) using a method described previously^7^ using HapMap 3 data as the reference panel, which includes 45 CHB (Han Chinese in Beijing, China) and 44 JPT (Japanese in Tokyo, Japan) individuals with 2 or 4-digit class I and class II classical allele types. In total, 118 *HLA* alleles, 3,972 SNPs, and 309 amino-acid positions were imputed including 229 biallelic positions and 71 positions with more than two amino acids. We performed the association analyses on the dosage data of all *HLA* alleles using the same linear regression model and the covariates as that for the SNP association analyses. Omnibus tests of amino acids with more than one allele were performed using the “--chap” command in PLINK.

## Additional Information

**How to cite this article**: Wei, Z. *et al.* Genome-Wide Association Studies of HIV-1 Host Control in Ethnically Diverse Chinese Populations. *Sci. Rep.* **5**, 10879; doi: 10.1038/srep10879 (2015).

## Supplementary Material

Supplementary Information

## Figures and Tables

**Table 1:** Phenotype and genotype characteristics of each cohort.

	**HAN**	**YUN**	**XIN**
Sample size	288	154	96
Ethnicity and sample sizes*	Han 274/Other 14	Han 52/Dai 58/Jingpo 40/Other 4	Uygur 83/Hui 11/Other 2
Mean age	35.3	34.6	33.9
Number of males/females	250/38	146/8	94/2
Mean viral load (and range) in log10	4.22 (2.38, 6.49)	4.28 (2.54, 5.87)	3.52 (1.30, 5.46)
Mean number of available viral-load points	2.1	1.4	1.0
Number of SNPs that passed quality control	481,717	488,154	528,294

^*^In the HAN group, patients of other ethnicities (“other”) included Manchu, Dai, Korean, Zhuang and Hui ethnicities. In the YUN group, these others included A’chang, De’ang and Lisu ethnicities. In the XIN group, the others included Dongxiang and Ozbek ethnicities.

**Table 2:** Replication of significant SNPs reported in Caucasian and African American populations.

					**HAN**			**YUN**			**XIN**			
**SNP**#	**Chr**	**Position**	**Alleles**	**Related gene**	**MAF**	**Beta**	***P*****value**	**MAF**	**Beta**	***P*****value**	**MAF**	**Beta**	***P*****value**	**Reference population**
rs2395029	6	31431780	C/A	*HCP5*	1.61%	0.1489	0.5229	0	—	—	6.11%	−0.0789	0.8112	Caucasian^6^,^7^,^27^
rs9264942*	6	31274380	G/A	*HLA-C*	48.21%	−0.1986	0.0018	44.30%	0.04828	0.6291	39.44%	0.1553	0.3233	Caucasian^6^,^7^,^27^
rs2523608	6	31322559	A/G	*HLA-B*	42.14%	−0.0319	0.6018	35.23%	−0.0282	0.7881	39.44%	−0.2181	0.1437	African American^7^
rs9262632	6	31024808	G/A	*HCG22*	5.00%	−0.1625	0.2885	15.10%	0.1649	0.2258	12.78%	0.174	0.4287	African American^7^
rs259919*	6	30025503	A/G	*ZNRD1-AS1*	23.93%	0.1627	0.0201	23.15%	0.1389	0.198	23.89%	−0.3281	0.0823	Caucasian^27^
rs9468692	6	30119890	A/C	*TRIM10*	6.61%	0.0016	0.9908	4.36%	−0.0735	0.7448	8.89%	0.2324	0.3828	Caucasian^27^
rs9266409*	6	31336568	G/A	*HLA-B*	34.82%	0.1516	0.0299	43.96%	−0.0588	0.5824	26.67%	0.1049	0.5211	Caucasian^27^
rs8192591	6	32185796	A/G	*NOTCH4*	2.14%	−0.0206	0.9273	0	—	—	4.44%	0.2773	0.4258	Caucasian^27^

“Chr” terms as chromosome; “MAF” terms as minor allele frequency.

^#^rs4418214, rs3131018 identified in a European population and rs2255221, rs2523590 identified in an African American population were not included in our chip.

^*^Significant SNPs in the HAN cohort.

**Table 3:** Association results of significant imputed HLA alleles.

**HLA allele**	**Allele frequency**	***P***value*	***P***_**condition**_
HLA-B*1302	9.30%	2.58 × 10^−5^	1.34 × 10^−2^
HLA-C*0602	13.60%	1.97 × 10^−4^	0.13

^*^P value of less than 2.50 × 10^−4^ is considered significant based on the Bonferroni correction.

*P*condition: *P* value of conditional test with rs2442719.

**Table 4:** Association results of imputed amino acids (*P* < 0.05).

**HLA-B**				**HLA-C**			
**Position**	**Alleles**	**Regions**	**P value**	**Position**	**Alleles**	**Regions**	**P value**
145	L/R	α2	6.24 × 10^−4^	156	L/W/R/D	α2	1.10 × 10^−4^
80	N/I/T	α1	9.12 × 10^−4^	73	A/T	α1	6.57 × 10^−4^
−8	V/L	Signal peptide	1.85 × 10^−3^	116	S/Y/L/F	α2	1.85 × 10^−3^
103	L/V	α2	2.09 × 10^−3^	219	W/R	α3	2.28 × 10^−3^
77	S/N/D	α1	3.51 × 10^−3^	90	D/A	α1	3.77 × 10^−3^
180	E/Q	α2	5.05 × 10^−3^	304	M/V	Transmembrane	3.80 × 10^−3^
177	D/E	α2	5.07 × 10^−3^	77	N/S	α1	4.54 × 10^−3^
178	K/T	α2	8.67 × 10^−3^	80	K/N	α1	4.55 × 10^−3^
71	A/T	α1	1.11 × 10^−2^	91	R/G	α2	4.59 × 10^−3^
41	T/A	α1	1.14 × 10^−2^	173	K/E	α2	8.21 × 10^−3^
95	L/W/I	α2	1.39 × 10^−2^	103	V/L	α2	8.90 × 10^−3^
−11	S/W	Signal peptide	1.61 × 10^−2^	95	L/I/F	α2	9.00 × 10^−3^
−16	L/V	Signal peptide	1.80 × 10^−2^	163	T/L	α2	1.07 × 10^−2^
147	H/Y	α2	1.80 × 10^−2^	94	I/T	α2	1.24 × 10^−2^
143	S/T	α2	1.81 × 10^−2^	1	G/C	α1	1.31 × 10^−2^
82	L/R	α1	2.01 × 10^−2^	9	D/Y/S/F	α1	1.64 × 10^−2^
83	R/G	α1	2.03 × 10^−2^	21	H/R	α1	2.27 × 10^−2^
81	A/L	α1	2.15 × 10^−2^	97	W/R	α2	2.77 × 10^−2^
152	E/V	α2	2.30 × 10^−2^				
74	D/Y	α1	3.83 × 10^−2^				
305	T/A	Transmembrane	3.86 × 10^−2^				
282	I/V	Connecting peptide	4.03 × 10^−2^				
245	T/A	α3	4.49 × 10^−2^				
