# Supplementary material for: Genome-Wide Association Studies of HIV-1 Host Control in Ethnically Diverse Chinese Populations
Source: Sci Rep. 2015 Jun 3;5:10879. doi: 10.1038/srep10879 (PMC4454153; doi:10.1038/srep10879)
Supplement: Supplementary Information [file srep10879-s1.pdf]

Supplementary Information

Genome-Wide Association Studies of HIV-1 Host Control in Ethnically Diverse Chinese Populations

Zejun Wei[1$], Yang Liu[1$], Heng Xu[2], Kun Tang[3], Hao Wu[4], Lin Lu[5], Zhe Wang[6], Zhengjie Chen[7], Junjie Xu[8], Yufei Zhu[1], Landian Hu[1], Hong Shang[8*], Guoping Zhao[9*], Xiangyin Kong[1*]

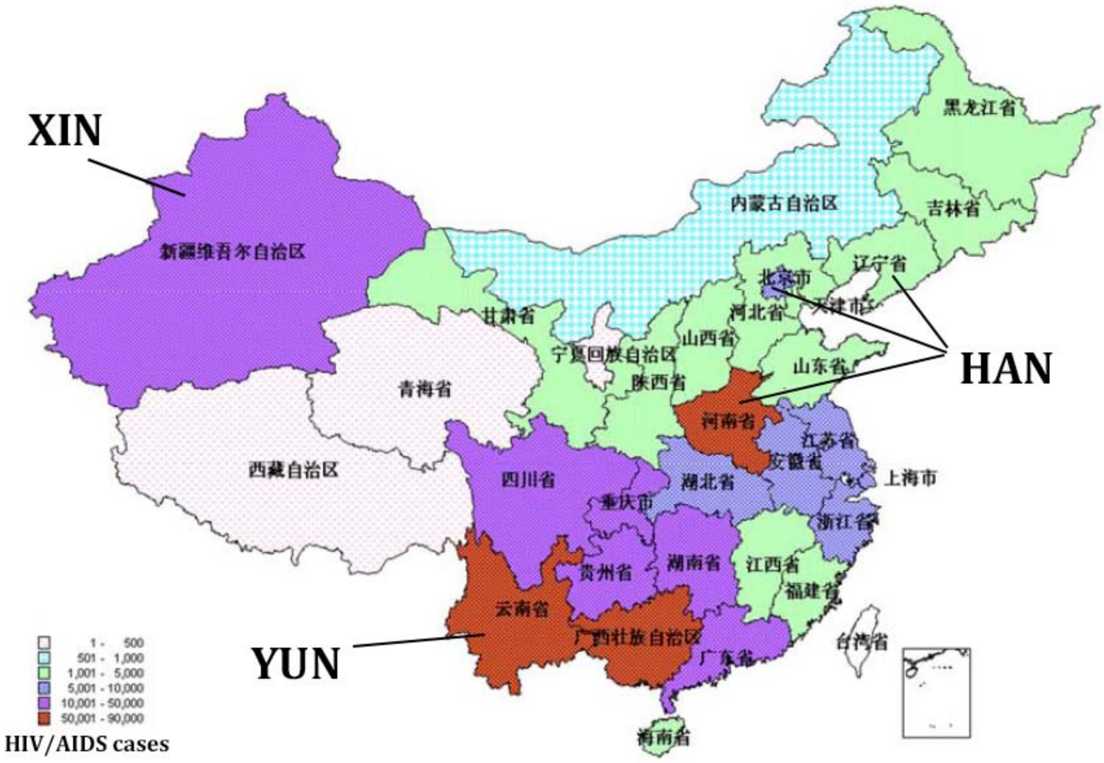

Supplementary Figure 1. **Geographical distribution of HAN, YUN, and XIN groups.**
We labeled HAN, YUN, and XIN groups on the map from "Figure 2 Geographical distribution of cumulative reported HIV/AIDS cases" in "2011 Report on the Estimation of HIV/AIDS Epidemic in China" (available at http://www.chinaaids.cn/fzdt/zxdd/201201/t20120129_1745902.htm).
Henan, Yunnan and Xinjiang province are regions with the highest numbers of reported HIV cases in China (as of 30th September 2011)[1]. Patients from the HAN group were from the Liaoning, Henan province and Beijing, which located in the center and north of China. Patients from the YUN group were from the Yunnan province in southwestern China. Patients of the XIN group were from the Xinjiang province in northwestern China. Xinjiang and Yunnan province are noted for their high levels of ethnic diversity in China [2].

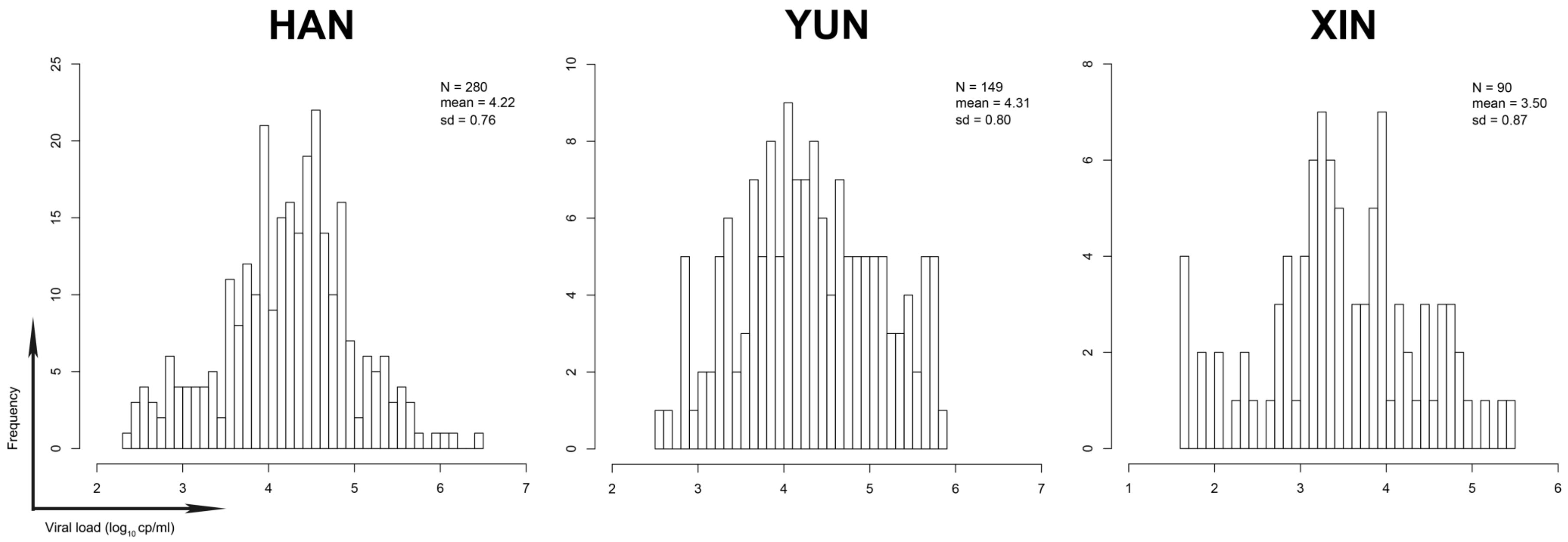

Supplementary Figure 2. **Viral-load distribution of the three groups.**

Patients numbers (y axis) were plotted against log_{10}(viral load). "N" terms as numbers of patients; "mean" terms as the mean values of log_{10}(viral load); "sd" terms as the standard deviation.

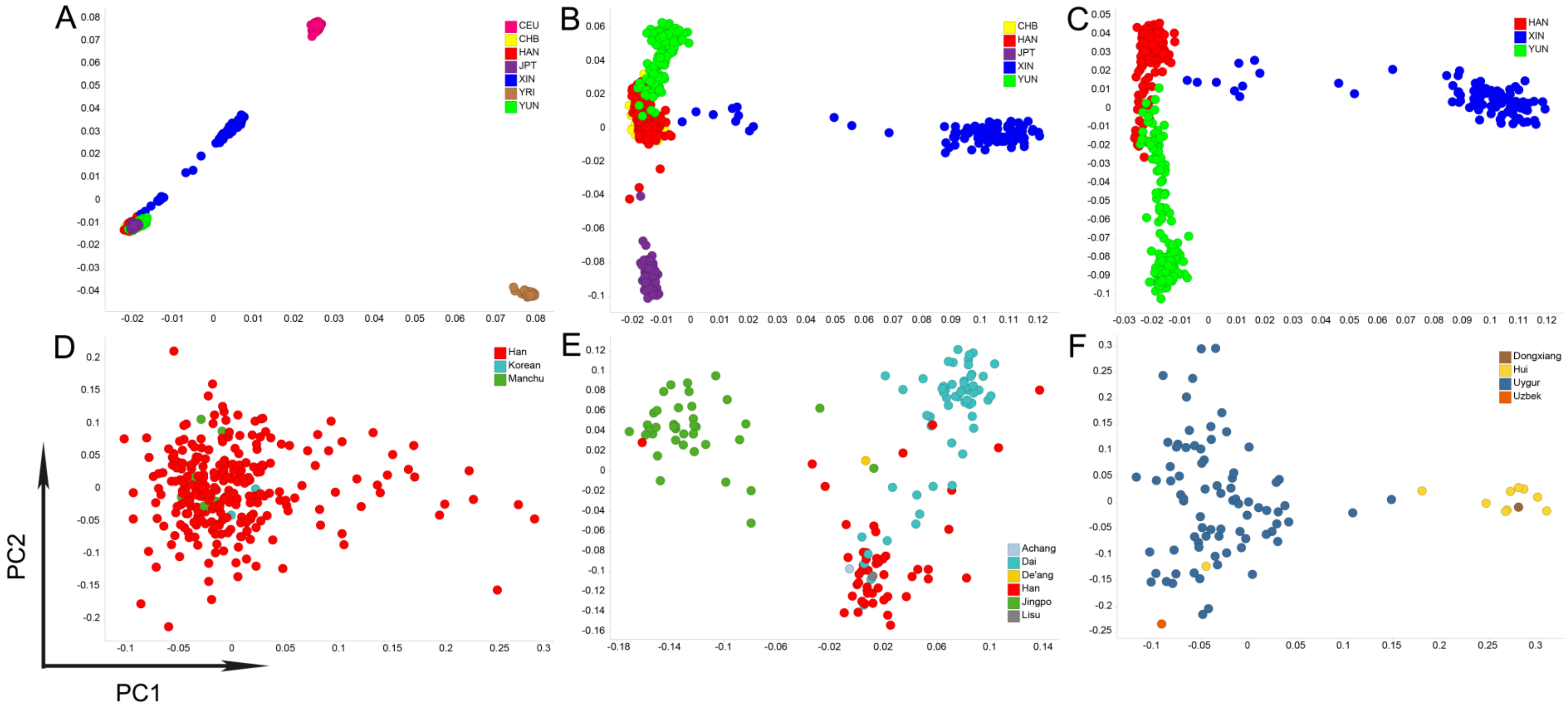

Supplementary Figure 3. The principle components analysis (PCA) of multi-ethnic patients of HAN (n=280), YUN (n=149), XIN (n=90) groups and reference samples from the HapMap.

Samples were plotted against the first two significant principle components (PC1 and PC2) derived from the PCA using EIGENSTRAT [3-5]. **(A)** Plots of 519 patients from HAN, YUN and XIN groups with 395 HapMap unrelated individuals including 112 Caucasians (CEU), 113 Africans (YRI), 86 Japanese (JPT), and 84 Han Chinese (CHB). HAN and YUN patients overlapped with CHB and JPT but separated from XIN. **(B)** Plots of Asians: YUN and XIN separated from HAN and CHB. **(C)** Plots of total patients: HAN, YUN and XIN separated from each other. **(D-E)** Plots of each group of patients with the different ethnicities labeled.

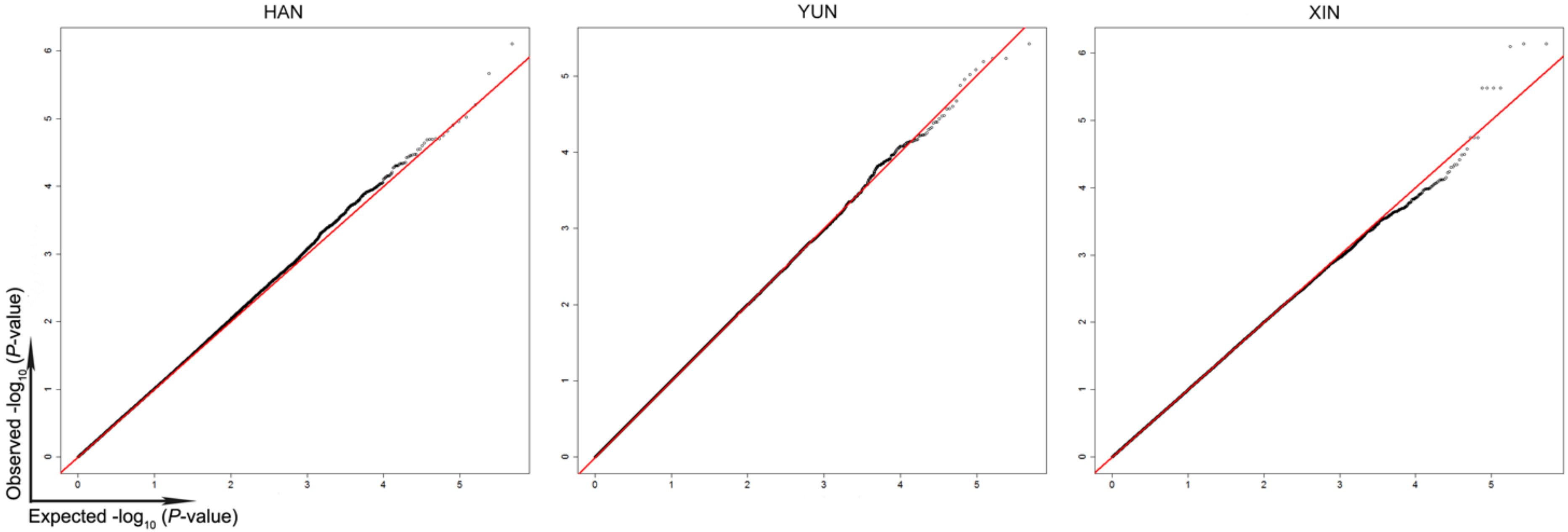

Supplementary Figure 4. Quantile-quantile (Q-Q) plot of linear regression analyses of the HAN, YUN, and XIN groups.

The negative logarithm of the observed (y-axis) and the expected (x-axis) *P* value is plotted for each SNP (dot), and the red line denotes the null hypothesis of no true association. Deviation from the expected p value distribution is evident only in the tail area (λ=1.01, 1.00, and 1.00 in HAN, YUN, and XIN, respectively) suggesting that population stratification was adequately controlled by adjusting for PCA.

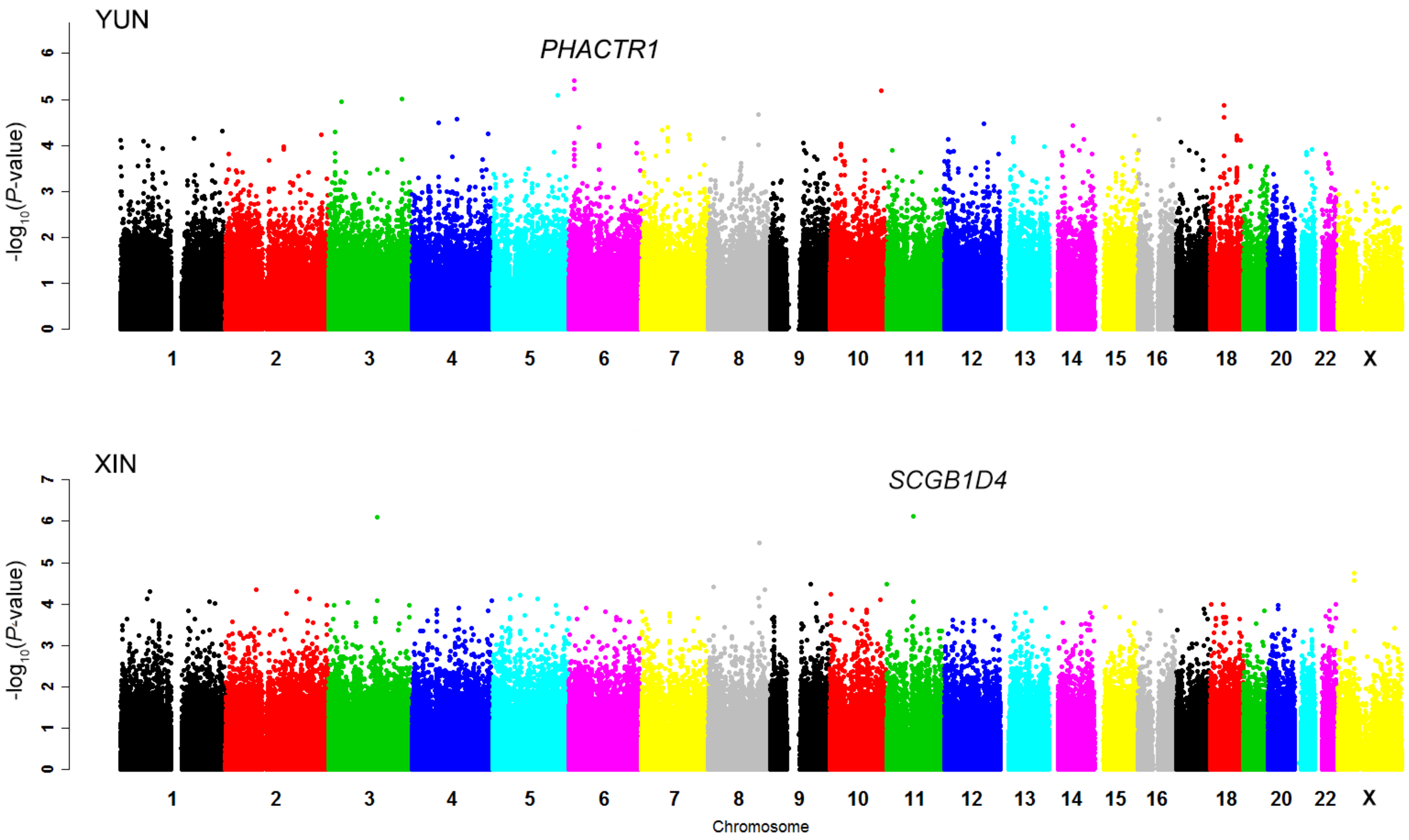

YUN
PHACTR1
-log10(P-value)
0
1
2
3
4
5
6
1 2 3 4 5 6 7 8 9 10 11 12 13 14 15 16 18 20 22 X
XIN
SCGB1D4
-log10(P-value)
0
1
2
3
4
5
6
7
1 2 3 4 5 6 7 8 9 10 11 12 13 14 15 16 18 20 22 X
Chromosome

Supplementary Figure 5. Manhattan plot for host control of HIV-1 outcome following infection in the YUN and XIN groups.

Association between genotype and HIV-1 viral-load set-point was evaluated using a linear regression model for 488,154 and 528,294 SNPs in YUN (N=149) and XIN (N=90) groups, respectively. *P* values ($-\log_{10} P$, y axis) were plotted against the respective chromosomal position of each SNP (x axis). Gene symbols are indicated for the top loci in each group.

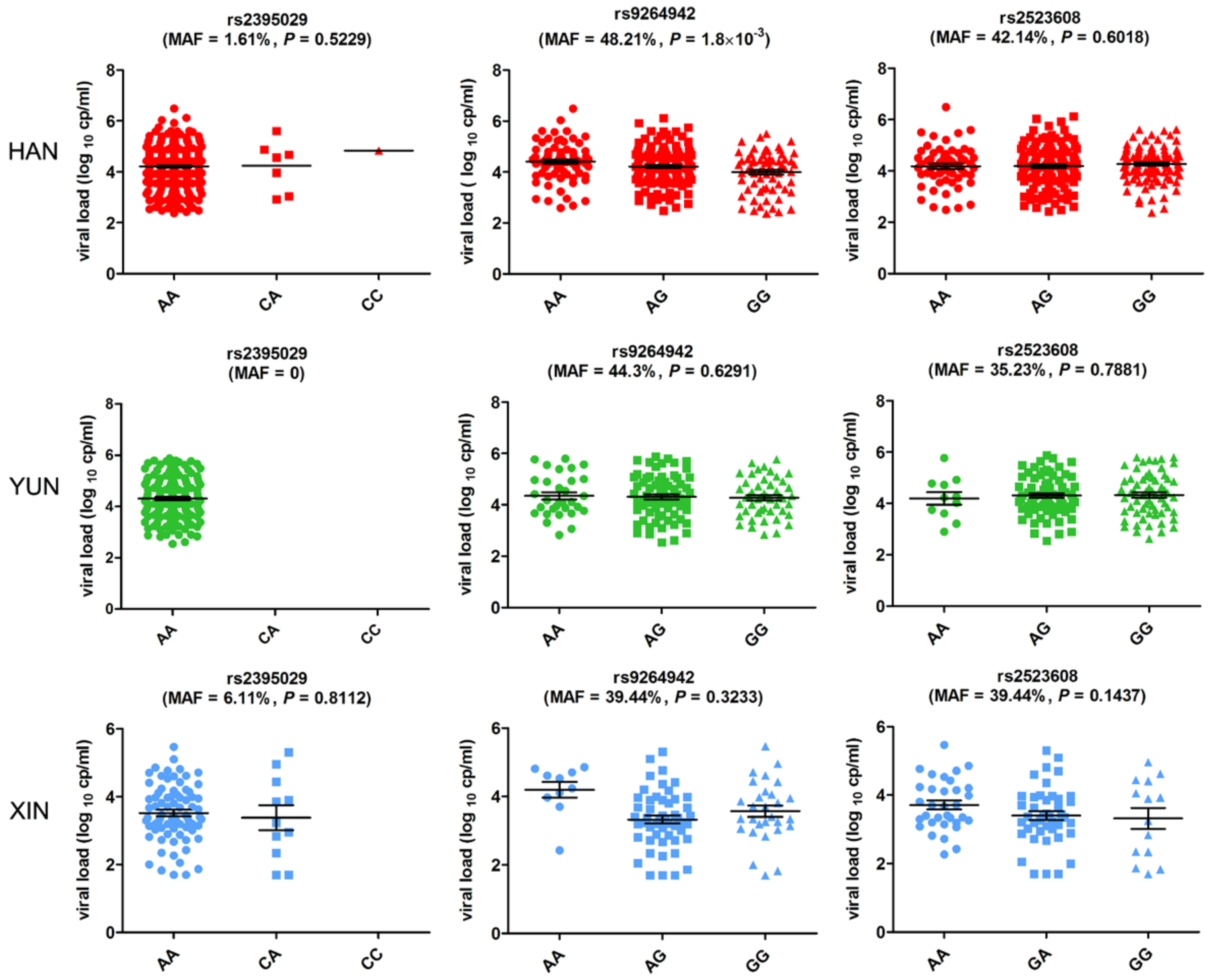

Supplementary Figure 6. Viral-load distribution at the reported SNPs in each group. Patients of HAN, YUN and XIN are labeled in red, green and blue, respectively. The black line denotes the mean value and the error bar represents the standard error of the mean. Log_{10}(viral load) (*y* axis) were plotted against patients with different alleles (x axis).

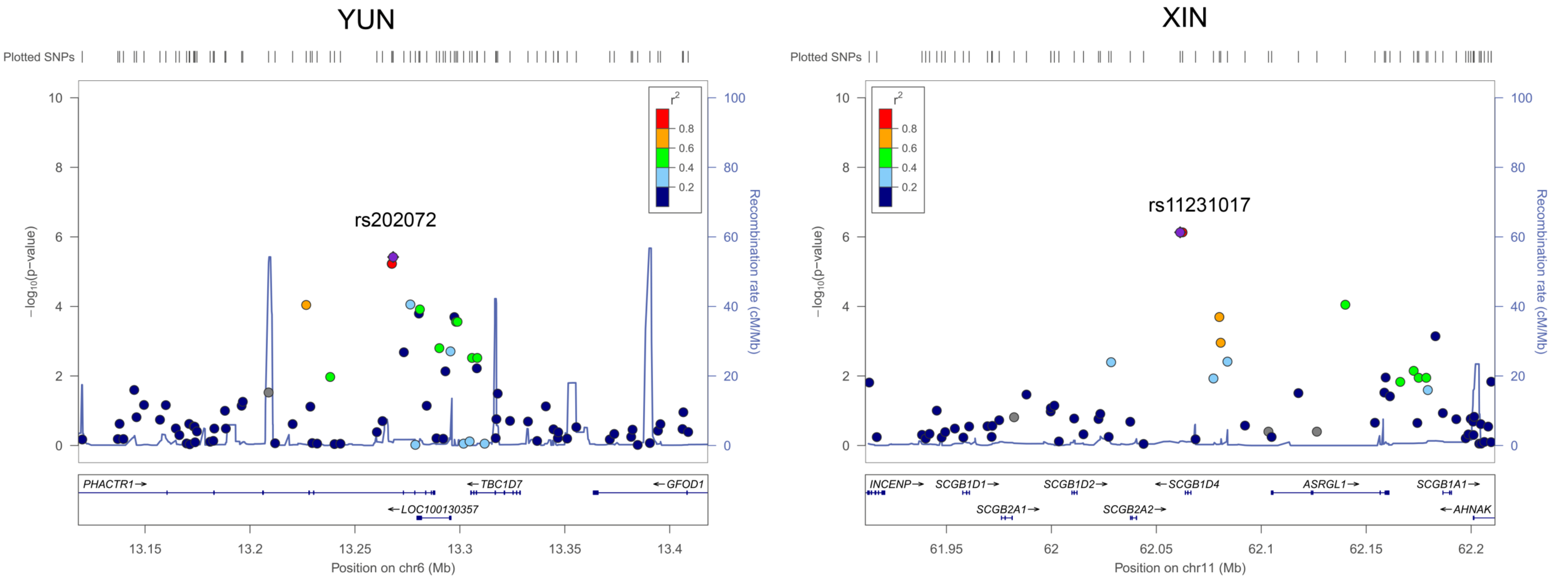

Supplementary Figure 7. Association results of SNPs around the top GWAS signal in the YUN and XIN groups.

Regional plots show association results for SNPs spanning Chr6: 13.1-13.4 Mb at *PHACTR1* and Chr11: 61.9-62.2 Mb at *SCGB1D4* in the YUN (left) and XIN (right) group, respectively. The plots were constructed using LocusZoom [6]: *P* values ($-\log_{10} P$, y axis) were plotted against respective chromosomal positions of each SNP (x-axis), and colors denote LD (r^2) with the top signal in 1000 genomes from East Asian populations.

Supplementary Table 1. Top association results in the HAN group ($P < 1\times10^{-4}$).

SNP	Chr	Position	Minor allele	MAF	Beta	*P* value	Gene	Gene Location
rs2442719	6	31320538	A	0.4161	-0.3298	7.85×10^{-7}	HLA-C | HLA-B	INTERGENIC
rs947612	6	73738661	A	0.2661	0.3459	2.15×10^{-6}	KCNQ5	INTRON
rs3763312	6	32376348	A	0.15	-0.4002	6.24×10^{-6}	BTNL2 | HLA-DRA	INTERGENIC
rs12210887	6	31815723	A	0.1232	-0.4137	9.53×10^{-6}	C6orf48 | NEU1	INTERGENIC
rs12528584	6	31011845	A	0.1679	-0.3638	1.09×10^{-5}	LOC729792	INTRON
rs13150924	4	170198810	A	0.0375	-0.7422	1.24×10^{-5}	SH3RF1 | NEK1	INTERGENIC
rs7680331	4	156540258	A	0.4643	0.2707	1.53×10^{-5}	LOC100287564 | GUCY1A3	INTERGENIC
rs2302291	17	67028260	G	0.2286	-0.3304	1.77×10^{-5}	ABCA9	CODING
rs3775771	4	70723053	C	0.01786	1.023	1.98×10^{-5}	SULT1E1	INTRON
rs6479245	9	107281379	A	0.2357	0.3219	1.99×10^{-5}	OR13F1 | OR13C4	INTERGENIC
rs10440778	5	124329306	G	0.4411	-0.2862	2.01×10^{-5}	ZNF608 | GRAMD3	INTERGENIC
rs3858668	12	23183392	A	0.3018	-0.2875	2.01×10^{-5}	ETNK1 | SOX5	INTERGENIC
rs7717298	5	16799341	G	0.2304	0.318	2.06×10^{-5}	MYO10	INTRON
rs2532924	6	30932682	G	0.3411	-0.2862	2.28×10^{-5}	DPCR1 | MUC21	INTERGENIC
rs3131296	6	32172993	A	0.09319	-0.4654	2.50×10^{-5}	NOTCH4	INTRON
rs1523666	9	107293424	A	0.2339	0.3142	2.82×10^{-5}	OR13C4 | OR13C3	INTERGENIC
rs3763309	6	32375973	A	0.1446	-0.3922	2.86×10^{-5}	BTNL2 | HLA-DRA	INTERGENIC
rs204999	6	32109979	G	0.1571	-0.3601	3.38×10^{-5}	FKBPL | PRRT1	INTERGENIC
rs6457730	6	33486732	A	0.02857	-0.8053	3.39×10^{-5}	ZBTB9 | BAK1	INTERGENIC
rs130065	6	31122500	A	0.1398	-0.37	3.45×10^{-5}	CCHCR1	CODING
rs10237037	7	7848087	A	0.03571	-0.7207	3.47×10^{-5}	LOC729852	INTRON
rs3134942	6	32168771	A	0.09107	-0.4714	3.55×10^{-5}	NOTCH4	CODING

rs8092957	18	3830606	C	0.04643	-0.6211	3.70×10^{-5}	DLGAP1	INTRON
rs11077922	17	67061807	A	0.3339	-0.272	3.76×10^{-5}	ABCA9 | ABCA6	INTERGENIC
rs10440752	5	124329101	C	0.4411	-0.2744	4.45×10^{-5}	ZNF608 | GRAMD3	INTERGENIC
rs6534743	4	130714768	A	0.2411	-0.2995	4.47×10^{-5}	C4orf33 | LOC100132483	INTERGENIC
rs9267546	6	31673436	A	0.1411	-0.3635	4.56×10^{-5}	BAT5 | LY6G6F	INTERGENIC
rs6481514	10	28520798	A	0.125	-0.3953	4.56×10^{-5}	MPP7 | LOC100288110	INTERGENIC
rs2815597	6	122991616	A	0.1196	-0.3974	4.57×10^{-5}	PKIB	INTRON
rs7215642	17	67027870	G	0.3	-0.291	4.80×10^{-5}	ABCA9	INTRON
rs3132580	6	30920124	A	0.09464	-0.4348	4.93×10^{-5}	DPCR1	CODING
rs17146892	X	37835168	A	0.2317	-0.4132	4.95×10^{-5}	DYNLT3 | CXorf27	INTERGENIC
rs11617918	13	99577147	A	0.3625	0.2687	4.96×10^{-5}	DOCK9	INTRON
rs3745651	19	12692001	A	0.06964	0.4954	4.96×10^{-5}	ZNF490	CODING
rs4678	6	30893941	A	0.1179	-0.399	5.22×10^{-5}	VARS2	CODING
rs2735076	6	29943490	A	0.2518	-0.2906	5.31×10^{-5}	HCG9	INTRON
rs2043356	10	127270143	A	0.2071	0.3166	6.11×10^{-5}	LOC100169752 | C10orf122	INTERGENIC
rs9351229	6	90629110	A	0.3482	0.2614	6.51×10^{-5}	GJA10 | BACH2	INTERGENIC
rs9363764	6	68232042	A	0.3214	0.276	6.65×10^{-5}	LOC100289348 | LOC100289683	INTERGENIC
rs7440685	4	156543095	A	0.4429	0.2513	6.82×10^{-5}	LOC100287564 | GUCY1A3	INTERGENIC
rs930443	16	60365853	A	0.2036	-0.298	6.99×10^{-5}	LOC644649 | LOC729159	INTERGENIC
rs7083854	10	127264189	G	0.2089	0.3137	7.01×10^{-5}	LOC100169752	INTRON
rs6841955	4	170212576	G	0.03571	-0.6936	7.04×10^{-5}	SH3RF1 | NEK1	INTERGENIC

rs13429217	2	49808562	A	0.1214	0.3682	7.16×10^{-5}	FSHR | NRXN1	INTERGENIC
rs1431034	14	41881857	A	0.3071	0.2719	7.36×10^{-5}	FBXO33 | LRFN5	INTERGENIC
rs4725031	7	7845273	A	0.03393	-0.705	7.46×10^{-5}	LOC729852	INTRON
rs2395163	6	32387809	G	0.1482	-0.3583	7.66×10^{-5}	BTNL2 | HLA-DRA	INTERGENIC
rs9513770	13	101259952	G	0.2054	0.3029	7.87×10^{-5}	TMTC4	INTRON
rs9961715	18	3824312	G	0.04821	-0.5868	8.70×10^{-5}	DLGAP1	INTRON
rs1155405	16	60376473	A	0.2125	-0.294	8.91×10^{-5}	LOC644649 | LOC729159	INTERGENIC
rs720465	6	31125777	A	0.1661	-0.3288	8.98×10^{-5}	CCHCR1	INTRON
rs1932011	10	55357136	A	0.07857	0.4748	9.04×10^{-5}	MBL2 | PCDH15	INTERGENIC
rs1489843	10	55367569	A	0.07857	0.4748	9.04×10^{-5}	MBL2 | PCDH15	INTERGENIC
rs6673363	1	30933736	A	0.09643	-0.4029	9.44×10^{-5}	LOC100288450 | MATN1	INTERGENIC
rs10915123	1	30940894	A	0.09643	-0.4029	9.44×10^{-5}	LOC100288450 | MATN1	INTERGENIC
rs1329917	9	17962477	C	0.4804	-0.2449	9.51×10^{-5}	SH3GL2 | ADAMTSL1	INTERGENIC
rs7798314	7	154472375	C	0.3536	0.2676	9.77×10^{-5}	DPP6	INTRON
rs4547755	4	162327265	G	0.2429	0.2806	9.88×10^{-5}	FSTL5	INTRON
rs754344	6	20243096	G	0.2768	0.2822	9.88×10^{-5}	MBOAT1 | E2F3	INTERGENIC

Chr: chromosome; MAF: minor allele frequency;

Supplementary Table 2. Replication of top significant 6p21.33 SNPs of the HAN group (P <1×10^{-4}) in the YUN and XIN groups.

			HAN				YUN				XIN			
SNP	Chr	Position	Minor Allele	MAF	Beta	P value	Minor Allele	MAF	Beta	P value	Minor Allele	MAF	Beta	P value
rs2442719	6	31320538	A	41.61%	-0.3298	7.85×10^{-7}	A	48.66%	0.02378	0.8149	G	39.44%	0.002281	0.9806
rs12210887	6	31815723	A	12.32%	-0.4137	9.53×10^{-6}	A	1.68%	-0.7541	0.05647	A	14.44%	-0.08454	0.64
rs12528584	6	31011845	A	16.79%	-0.3638	1.09×10^{-5}	A	7.38%	-0.2112	0.2723	A	26.11%	-0.1095	0.433
rs2532924	6	30932682	G	34.11%	-0.2862	2.28×10^{-5}	G	40.27%	0.007878	0.9398	A	48.89%	-0.06058	0.6365
*rs130065	6	31122500	A	13.98%	-0.37	3.45×10^{-5}	A	2.01%	-0.7685	0.01046	A	18.89%	0.04029	0.8763
rs9267546	6	31673436	A	14.11%	-0.3635	4.56×10^{-5}	A	8.73%	-0.08129	0.6319	A	15.56%	-0.1473	0.411
*rs3132580	6	30920124	A	9.46%	-0.4348	4.93×10^{-5}	A	1.68%	-0.9708	0.01308	A	7.78%	-0.02302	0.8225
rs4678	6	30893941	A	11.79%	-0.399	5.22×10^{-5}	A	2.69%	-0.4767	0.119	A	14.44%	0.2532	0.2826
rs720465	6	31125777	A	16.61%	-0.3288	8.98×10^{-5}	A	3.69%	-0.4495	0.09757	A	28.89%	-0.3163	0.05967

*significant SNPs replicated in YUN group.

Chr: chromosome; MAF: minor allele frequency;

Supplementary Table 3. Top association results in the YUN group ($P < 1\times10^{-4}$).

SNP	Chr	Position	Minor allele	MAF	Beta	*P* value	Gene	Gene Location
rs202072	6	13268211	A	0.4497	-0.4038	3.80×10^{-6}	PHACTR1	INTRON
rs182148	6	13267547	G	0.4396	-0.3971	5.88×10^{-6}	PHACTR1	INTRON
rs366904	6	13267681	A	0.4396	-0.3971	5.88×10^{-6}	PHACTR1	INTRON
rs2420903	10	122802506	A	0.3691	-0.4386	6.48×10^{-6}	BRWD2 | LOC100129699	INTERGENIC
rs460626	5	154437261	G	0.07718	0.7899	8.26×10^{-6}	KIF4B | SGCD	INTERGENIC
rs1948161	3	173491396	G	0.4128	0.3947	9.60×10^{-6}	NLGN1	INTRON
rs4955188	3	31273355	G	0.1376	0.533	1.11×10^{-5}	GADL1 | STT3B	INTERGENIC
rs503301	18	32563337	A	0.2584	0.4366	1.32×10^{-5}	MAPRE2	INTRON
rs11783511	8	118826698	G	0.4396	0.3997	2.14×10^{-5}	EXT1	INTRON
rs1424451	18	32550479	C	0.2685	0.4325	2.49×10^{-5}	DTNA | MAPRE2	INTERGENIC
rs4785434	16	50587976	A	0.302	0.436	2.67×10^{-5}	NKD1	INTRON
rs1522207	4	107420809	A	0.3523	0.3917	2.73×10^{-5}	LOC100288276	INTRON
rs1397546	4	62915024	G	0.2248	0.4896	3.28×10^{-5}	LPHN3	INTRON
rs10859481	12	93706256	A	0.4463	-0.4046	3.35×10^{-5}	LOC100287580 | NUDT4	INTERGENIC
rs1152505	14	56824215	A	0.4396	0.3978	3.63×10^{-5}	PELI2 | C14orf101	INTERGENIC
rs11759284	6	24494517	C	0.2919	-0.4125	4.05×10^{-5}	GPLD1 | ALDH5A1	INTERGENIC
rs4646828	6	24494924	A	0.2919	-0.4125	4.05×10^{-5}	GPLD1 | ALDH5A1	INTERGENIC
rs4718012	7	63618161	G	0.3255	0.3986	4.13×10^{-5}	ZNF727 | ZNF735	INTERGENIC
rs17799219	7	51137856	A	0.2919	0.4189	4.73×10^{-5}	COBL	INTRON
rs1025599	1	242297876	G	0.3221	-0.4019	4.86×10^{-5}	PLD5	INTRON

rs574141	3	16405740	A	0.245	0.4459	5.02×10^{-5}	RFTN1	INTRON
rs4862035	4	179614998	G	0.2315	0.4327	5.54×10^{-5}	LOC285501 | LOC100288304	INTERGENIC
rs10500030	7	113091026	G	0.06376	-0.8227	5.86×10^{-5}	LOC401397 | LOC100287664	INTERGENIC
rs10498183	2	226174116	A	0.1275	0.543	5.91×10^{-5}	DOCK10 | KIAA1486	INTERGENIC
rs4673141	2	226191730	A	0.1275	0.543	5.91×10^{-5}	DOCK10 | KIAA1486	INTERGENIC
rs2009296	18	63523971	A	0.08054	-0.7089	6.02×10^{-5}	CDH7	INTRON
rs2291343	18	63530016	A	0.08054	-0.7089	6.02×10^{-5}	CDH7	CODING
rs1031912	15	94590228	G	0.09459	-0.6136	6.17×10^{-5}	UNQ9370 | MCTP2	INTERGENIC
rs3011621	13	30079484	G	0.03356	-0.9303	6.81×10^{-5}	KIAA0774	UTR
rs2628202	18	63536167	A	0.0604	-0.8051	6.83×10^{-5}	CDH7	INTRON
rs1484700	18	63541240	G	0.0604	-0.8051	6.83×10^{-5}	CDH7	INTRON
rs12082376	1	175606606	A	0.08389	0.6987	6.99×10^{-5}	TNR	INTRON
rs1583721	7	63444577	A	0.3591	0.3712	7.12×10^{-5}	LOC100129126 | ZNF727	INTERGENIC
rs6995839	8	36953680	A	0.02349	1.226	7.17×10^{-5}	KCNU1 | ZNF703	INTERGENIC
rs12667497	7	115956129	G	0.4631	-0.3526	7.20×10^{-5}	TES | CAV2	INTERGENIC
rs2178166	7	115968690	G	0.4631	-0.3526	7.20×10^{-5}	TES | CAV2	INTERGENIC
rs9323719	14	82669795	G	0.2081	-0.4787	7.37×10^{-5}	SEL1L | FLRT2	INTERGENIC
rs11048434	12	9153932	A	0.3758	0.4031	7.39×10^{-5}	KLRG1	INTRON
rs9961146	18	62950676	G	0.04362	-0.912	7.52×10^{-5}	C18orf20 | CDH7	INTERGENIC
rs6566162	18	62956303	G	0.04362	-0.912	7.52×10^{-5}	C18orf20 | CDH7	INTERGENIC
rs260512	1	2182470	G	0.104	0.6379	7.65×10^{-5}	SKI	INTRON
rs8090208	18	71643807	A	0.08054	0.6391	7.80×10^{-5}	LOC100289370 |	INTERGENIC

							FBXO15	
rs11980921	7	63704031	G	0.4698	-0.3515	7.93×10^{-5}	ZNF679	INTRON
rs17113021	1	56342625	C	0.4899	-0.3542	8.19×10^{-5}	USP24 | LOC100288320	INTERGENIC
rs11078017	17	11235162	A	0.3624	-0.3806	8.33×10^{-5}	FLJ45455	INTRON
rs2482087	13	30084173	G	0.0302	-0.9553	8.40×10^{-5}	SLC7A1	UTR
rs3013641	13	30088049	T	0.0302	-0.9553	8.40×10^{-5}	SLC7A1	UTR
rs2482089	13	30091714	G	0.0302	-0.9553	8.40×10^{-5}	SLC7A1	CODING
rs2482090	13	30091819	G	0.0302	-0.9553	8.40×10^{-5}	SLC7A1	CODING
rs7867316	9	79519243	A	0.2315	0.4247	8.67×10^{-5}	PRUNE2	INTRON
rs1512414	6	13276415	A	0.396	-0.3619	8.72×10^{-5}	PHACTR1	INTRON
rs2013600	6	160370811	A	0.4463	0.362	8.75×10^{-5}	MAS1 | IGF2R	INTERGENIC
rs1232381	6	13226792	A	0.3826	-0.3618	9.01×10^{-5}	PHACTR1	INTRON
rs2367979	10	25980557	G	0.104	0.6255	9.20×10^{-5}	GPR158 | MYO3A	INTERGENIC
rs4370563	8	118823626	A	0.4329	0.3707	9.55×10^{-5}	EXT1	INTRON
rs550675	6	70979193	A	0.3221	-0.3995	9.81×10^{-5}	COL9A1	INTRON

Chr: chromosome; MAF: minor allele frequency;

Supplementary Table 4. Top association results in the XIN group (*P* < 1×10⁻⁴).

SNP	Chr	Position	Minor allele	MAF	Beta	*P* value	Gene	Gene Location
rs11231017	11	62061349	A	0.3111	-0.7189	7.39×10^{-7}	SCGB2A2 | SCGB1D4	INTERGENIC
rs11231018	11	62062509	G	0.3111	-0.7189	7.39×10^{-7}	SCGB2A2 | SCGB1D4	INTERGENIC
rs16823858	3	115278682	A	0.06667	-1.294	8.11×10^{-7}	ZBTB20 |	INTERGENIC
rs7836436	8	121006828	G	0.05	-1.401	3.31×10^{-6}	DEPDC6	INTRON
rs4501620	8	121013590	G	0.05	-1.401	3.31×10^{-6}	DEPDC6	INTRON
rs7820945	8	121016526	C	0.05	-1.401	3.31×10^{-6}	DEPDC6	INTRON
rs7835799	8	121042410	A	0.05	-1.401	3.31×10^{-6}	DEPDC6	INTRON
rs3761594	23	40964127	A	0.1429	-1.151	1.83×10^{-5}	USP9X	INTRON
rs2267494	23	41049864	G	0.1429	-1.151	1.83×10^{-5}	USP9X	INTRON
rs2284116	23	41051780	C	0.1429	-1.151	1.83×10^{-5}	USP9X	INTRON
rs2109472	23	41001048	C	0.1538	-1.09	2.67×10^{-5}	USP9X	INTRON
rs6479466	9	95958095	G	0.3278	-0.5998	3.24×10^{-5}	WNK2	INTRON
rs11246126	11	362668	A	0.2722	0.6698	3.30×10^{-5}	IFITM3 | B4GALNT4	INTERGENIC
rs1671400	8	13176407	A	0.08333	1.045	3.88×10^{-5}	DLC1	INTRON
rs4852808	2	71812455	G	0.2611	-0.6518	4.60×10^{-5}	DYSF	INTRON
rs7822343	8	134463161	C	0.02778	-1.701	4.60×10^{-5}	NDRG1 | ST3GAL1	INTERGENIC
rs9326089	1	69909410	A	0.4722	-0.679	4.88×10^{-5}	DEPDC1 | LRRC7	INTERGENIC
rs4629128	2	169173631	A	0.1389	0.8669	4.97×10^{-5}	STK39 | LASS6	INTERGENIC
rs17131865	10	3230337	A	0.05556	1.178	5.77×10^{-5}	PITRM1 | LOC100287172	INTERGENIC
rs6886919	5	64709775	C	0.08889	-0.9684	6.01×10^{-5}	ADAMTS6	INTRON
rs9297586	8	119427347	A	0.2278	0.6428	7.09×10^{-5}	SAMD12	INTRON

rs10497796	2	197806241	C	0.03333	-1.504	7.57×10^{-5}	PGAP1 | ANKRD44	INTERGENIC
rs7725810	5	40827307	G	0.3056	0.5759	7.61×10^{-5}	PRKAA1 | RPL37	INTERGENIC
rs10515378	5	107129158	G	0.08333	-1.025	7.61×10^{-5}	EFNA5 | FBXL17	INTERGENIC
rs12119690	1	63484437	A	0.1278	-0.7626	7.66×10^{-5}	ATG4C | FOXD3	INTERGENIC
rs2253812	10	119679259	A	0.07778	-0.9428	7.88×10^{-5}	EMX2 | RAB11FIP2	INTERGENIC
rs16823850	3	115274499	G	0.07778	-0.9205	8.20×10^{-5}	ZBTB20 |	INTERGENIC
rs12642793	4	190091809	C	0.1222	0.8038	8.49×10^{-5}	TRIML1 | FRG1	INTERGENIC
rs4951543	1	210885868	G	0.2333	-0.6248	8.76×10^{-5}	KCNH1	INTRON
rs10792346	11	62140069	A	0.3556	-0.5424	8.92×10^{-5}	ASRGL1	INTRON
rs9311384	3	46103974	A	0.1056	-0.8503	9.28×10^{-5}	XCR1 | CCR1	INTERGENIC
rs2001890	1	224494670	A	0.4944	0.5415	9.57×10^{-5}	NVL	INTRON
rs328012	9	108037717	C	0.15	0.7177	9.70×10^{-5}	SLC44A1	INTRON

Chr: chromosome; MAF: minor allele frequency;

Supplementary Table 5. Replication of novel SNPs between the three groups.

			HAN				YUN				XIN			
SNP	Chr	Position	Minor Allele	MAF	Beta	*P* value	Minor Allele	MAF	Beta	*P* value	Minor Allele	MAF	Beta	*P* value
rs947612	6	73738661	A	26.61%	0.3459	2.15×10^{-6}	A	23.49%	0.1092	0.3076	G	40.56%	-0.01315	0.9247
rs11231018	11	62062509	G	25.00%	-0.01005	0.8952	G	25.84%	0.1026	0.3377	G	31.11%	-0.7189	7.39×10^{-7}
rs202072	6	13268211	G	46.25%	0.06415	0.3437	A	44.97%	-0.4038	3.80×10^{-6}	A	32.78%	-0.01859	0.8949

Chr: chromosome; MAF: minor allele frequency;

Supplementary Table 6 Transethnic meta analysis results [7]. (Log_{10} BF ≥ 3)

								HAN				XIN				YUN			
SNP	Chr	Position	A1	A2	Log_{10} BF	$P_{posterior}$	Direction	Freq	Beta	SE	P	Freq	Beta	SE	P	Freq	Beta	SE	P
rs11231017	11	62061349	A	G	5.41	1	--+	0.248	-0.016	0.077	8.40E-01	0.311	-0.719	0.134	7.39E-07	0.258	0.103	0.107	3.38E-01
rs11231018	11	62062509	G	A	5.40	1	--+	0.250	-0.010	0.076	8.95E-01	0.311	-0.719	0.134	7.39E-07	0.258	0.103	0.107	3.38E-01
rs16823858	3	115278682	A	G	5.32	1	+--	0.097	0.084	0.115	4.68E-01	0.067	-1.294	0.242	8.11E-07	0.128	-0.003	0.144	9.85E-01
rs941349	3	22110442	C	A	5.23	0.16	---	0.379	-0.227	0.068	8.97E-04	0.367	-0.400	0.142	6.03E-03	0.453	-0.278	0.093	3.29E-03
rs10237037	7	7848087	A	G	4.68	0.238	---	0.036	-0.721	0.171	3.47E-05	0.044	-0.586	0.267	3.12E-02	0.057	-0.394	0.211	6.39E-02
rs9931202	16	6573408	A	C	4.56	0.161	---	0.405	-0.255	0.065	1.21E-04	0.311	-0.290	0.147	5.15E-02	0.376	-0.188	0.090	3.78E-02
rs720465	6	31125777	A	C	4.31	0.182	---	0.166	-0.329	0.083	8.98E-05	0.289	-0.322	0.168	5.97E-02	0.037	-0.449	0.269	9.76E-02
rs3763309	6	32375973	A	C	4.30	0.176	---	0.145	-0.392	0.092	2.86E-05	0.233	-0.221	0.181	2.26E-01	0.070	-0.309	0.181	8.96E-02
rs12210887	6	31815723	A	C	4.27	0.289	---	0.123	-0.414	0.092	9.53E-06	0.144	-0.102	0.218	6.40E-01	0.017	-0.729	0.379	5.65E-02
rs1557033	9	124242016	G	A	4.12	0.183	---	0.259	-0.234	0.070	9.75E-04	0.211	-0.439	0.155	5.77E-03	0.218	-0.200	0.113	7.87E-02
rs4725031	7	7845273	A	G	4.04	0.225	---	0.034	-0.705	0.175	7.46E-05	0.039	-0.534	0.311	8.92E-02	0.060	-0.370	0.206	7.50E-02
rs6478510	9	124227499	A	G	4.02	0.16	---	0.245	-0.261	0.072	3.58E-04	0.200	-0.392	0.158	1.51E-02	0.188	-0.191	0.125	1.29E-01
rs2251494	6	74818731	A	G	3.80	0.973	+-+	0.413	0.217	0.067	1.29E-03	0.400	-0.347	0.148	2.11E-02	0.373	0.294	0.090	1.34E-03
rs754344	6	20243096	G	A	3.79	0.137	+++	0.277	0.282	0.071	9.88E-05	0.322	0.191	0.140	1.77E-01	0.289	0.193	0.104	6.46E-02
rs3761594	X	40964127	A	G	3.78	1	+-+	0.158	0.171	0.117	1.45E-01	0.143	-1.151	0.252	1.83E-05	0.235	0.051	0.156	7.43E-01
rs12528584	6	31011845	A	G	3.72	0.205	---	0.168	-0.364	0.081	1.09E-05	0.261	-0.127	0.161	4.33E-01	0.074	-0.211	0.192	2.72E-01
rs2295133	14	97594218	A	G	3.71	0.159	---	0.488	-0.206	0.067	2.38E-03	0.400	-0.208	0.136	1.28E-01	0.503	-0.273	0.095	4.86E-03
rs2284116	X	41051780	C	A	3.59	1	+-+	0.152	0.137	0.119	2.53E-01	0.143	-1.151	0.252	1.83E-05	0.229	0.029	0.159	8.54E-01
rs4291067	6	74832124	G	A	3.58	0.979	+-+	0.413	0.217	0.067	1.29E-03	0.400	-0.347	0.148	2.11E-02	0.373	0.294	0.090	1.34E-03
rs947612	6	73738661	A	G	3.57	0.35	+++	0.266	0.346	0.071	2.15E-06	0.594	0.013	0.139	9.25E-01	0.235	0.109	0.107	3.08E-01
rs1897115	2	26554063	A	G	3.52	0.174	---	0.230	-0.257	0.072	4.15E-04	0.689	-0.068	0.166	6.82E-01	0.379	-0.252	0.099	1.21E-02
rs3134942	6	32168771	A	C	3.49	0.249	---	0.091	-0.471	0.112	3.55E-05	0.117	-0.101	0.236	6.69E-01	0.057	-0.341	0.206	1.01E-01
rs11042318	11	9394830	A	G	3.46	0.186	+++	0.075	0.346	0.119	4.07E-03	0.072	0.436	0.268	1.08E-01	0.077	0.474	0.174	7.21E-03
rs894220	1	30183014	A	G	3.44	0.169	+++	0.304	0.279	0.074	2.07E-04	0.283	0.250	0.172	1.48E-01	0.282	0.167	0.103	1.05E-01

rs1403544	15	37214431	G	A	3.42	0.154	---	0.289	-0.256	0.069	2.24E-04	0.367	-0.243	0.135	7.53E-02	0.265	-0.138	0.110	2.12E-01
rs130065	6	31122500	A	G	3.41	0.495	-+-	0.140	-0.370	0.088	3.45E-05	0.189	0.029	0.184	8.76E-01	0.020	-0.757	0.292	1.05E-02
rs855203	12	102958073	C	A	3.38	0.304	---	0.036	-0.420	0.166	1.19E-02	0.089	-0.215	0.210	3.10E-01	0.054	-0.733	0.205	4.79E-04
rs1264350	6	30796545	G	A	3.37	0.314	---	0.095	-0.398	0.106	2.03E-04	0.072	-0.062	0.295	8.33E-01	0.017	-0.940	0.374	1.31E-02
rs3129975	6	30732054	G	A	3.33	0.156	---	0.170	-0.303	0.081	2.45E-04	0.217	-0.149	0.183	4.20E-01	0.158	-0.259	0.137	6.05E-02
rs6479245	9	107281379	A	G	3.33	0.269	+++	0.236	0.322	0.074	1.99E-05	0.378	0.211	0.138	1.31E-01	0.258	0.050	0.105	6.30E-01
rs2395163	6	32387809	G	A	3.31	0.191	---	0.148	-0.358	0.089	7.66E-05	0.272	-0.140	0.177	4.32E-01	0.074	-0.259	0.178	1.48E-01
rs7300093	12	26746089	A	G	3.30	0.147	---	0.238	-0.197	0.074	7.71E-03	0.494	-0.299	0.145	4.19E-02	0.208	-0.301	0.118	1.16E-02
rs13213559	6	122979819	C	A	3.29	0.285	---	0.095	-0.420	0.110	1.65E-04	0.117	-0.006	0.197	9.78E-01	0.181	-0.290	0.120	1.66E-02
rs2501180	6	74828682	G	A	3.28	0.99	-+-	0.475	-0.210	0.065	1.46E-03	0.539	0.389	0.143	8.07E-03	0.524	-0.226	0.094	1.75E-02
rs9668898	12	26747260	A	G	3.28	0.149	---	0.238	-0.197	0.074	7.71E-03	0.494	-0.299	0.145	4.19E-02	0.208	-0.301	0.118	1.16E-02
rs2442719	6	31320538	A	G	3.28	0.69	-++	0.416	-0.330	0.065	7.85E-07	0.606	0.004	0.158	9.81E-01	0.487	0.024	0.100	8.15E-01
rs3132580	6	30920124	A	G	3.25	0.342	---	0.095	-0.435	0.105	4.93E-05	0.078	-0.064	0.284	8.23E-01	0.017	-0.940	0.374	1.31E-02
rs2047176	5	36056800	A	G	3.25	0.2	-+-	0.363	-0.253	0.065	1.19E-04	0.528	0.005	0.142	9.71E-01	0.336	-0.203	0.099	4.13E-02
rs3131296	6	32172993	A	G	3.24	0.266	---	0.093	-0.465	0.109	2.50E-05	0.117	-0.101	0.236	6.69E-01	0.057	-0.341	0.206	1.01E-01
rs1490813	5	67139113	G	A	3.23	0.239	+++	0.414	0.177	0.066	7.90E-03	0.533	0.415	0.134	2.59E-03	0.473	0.170	0.097	8.23E-02
rs13205342	6	122985277	A	G	3.23	0.278	---	0.095	-0.420	0.110	1.65E-04	0.117	-0.006	0.197	9.78E-01	0.181	-0.290	0.120	1.66E-02
rs11077922	17	67061807	A	C	3.22	0.454	--+	0.334	-0.272	0.065	3.76E-05	0.367	-0.329	0.140	2.14E-02	0.373	0.005	0.099	9.58E-01
rs10894121	11	129602247	A	G	3.20	0.186	---	0.146	-0.317	0.089	4.57E-04	0.167	-0.299	0.179	9.83E-02	0.084	-0.262	0.173	1.32E-01
rs1405043	10	108149479	G	A	3.19	0.199	+++	0.118	0.317	0.097	1.22E-03	0.067	0.118	0.287	6.82E-01	0.117	0.364	0.137	8.94E-03
rs10943076	6	73727741	A	G	3.19	0.166	+++	0.330	0.248	0.070	4.95E-04	0.506	0.125	0.133	3.53E-01	0.279	0.209	0.099	3.70E-02
rs494620	6	31838713	A	G	3.16	0.139	---	0.443	-0.165	0.062	8.44E-03	0.478	-0.234	0.135	8.64E-02	0.366	-0.288	0.099	4.33E-03
rs13358038	5	76404051	A	G	3.16	1	-+-	0.163	-0.196	0.088	2.73E-02	0.183	0.645	0.165	1.89E-04	0.188	-0.161	0.127	2.08E-01
rs503301	18	32563337	A	G	3.10	0.617	+++	0.248	0.130	0.072	7.25E-02	0.222	0.017	0.155	9.12E-01	0.258	0.437	0.097	1.32E-05
rs202072	6	13268211	G	A	3.09	0.86	+++	0.463	0.064	0.068	3.44E-01	0.672	0.019	0.140	8.95E-01	0.550	0.404	0.084	3.80E-06
rs6435829	2	215361680	A	G	3.09	0.177	---	0.450	-0.248	0.063	1.18E-04	0.472	-0.088	0.141	5.35E-01	0.530	-0.145	0.100	1.50E-01
rs4507103	2	215361613	A	G	3.07	0.164	---	0.450	-0.248	0.063	1.18E-04	0.472	-0.088	0.141	5.35E-01	0.530	-0.145	0.100	1.50E-01

rs883115	1	224807561	A	G	3.07	0.241	---	0.200	-0.181	0.078	2.20E-02	0.289	-0.227	0.151	1.37E-01	0.178	-0.425	0.122	6.31E-04
rs132210	22	48143913	G	A	3.06	0.182	+++	0.334	0.159	0.069	2.26E-02	0.489	0.372	0.142	1.04E-02	0.386	0.231	0.095	1.63E-02
rs586382	5	36052919	G	A	3.06	0.229	-+-	0.371	-0.249	0.064	1.29E-04	0.528	0.005	0.142	9.71E-01	0.352	-0.186	0.097	5.80E-02
rs13199524	6	32066765	A	G	3.04	0.304	-+-	0.114	-0.390	0.099	1.05E-04	0.167	0.017	0.206	9.36E-01	0.054	-0.386	0.213	7.21E-02
rs11256534	10	10257013	A	G	3.03	0.211	---	0.030	-0.636	0.188	8.12E-04	0.056	-0.521	0.313	1.00E-01	0.013	-0.629	0.415	1.32E-01
rs9267546	6	31673436	A	G	3.01	0.229	---	0.141	-0.364	0.088	4.56E-05	0.156	-0.160	0.194	4.11E-01	0.087	-0.081	0.169	6.32E-01
rs7858074	9	128883849	A	G	3.01	0.303	+++	0.273	0.267	0.069	1.28E-04	0.222	0.356	0.155	2.43E-02	0.312	0.030	0.111	7.89E-01
rs12233901	4	85360873	A	G	3.01	0.275	+++	0.082	0.393	0.122	1.41E-03	0.078	0.652	0.239	7.82E-03	0.097	0.185	0.161	2.52E-01
rs2395157	6	32348145	G	A	3.01	0.211	---	0.177	-0.344	0.087	1.03E-04	0.283	-0.302	0.173	8.54E-02	0.101	-0.078	0.156	6.19E-01
rs12750699	1	167273760	C	A	3.01	0.232	---	0.123	-0.349	0.097	3.79E-04	0.111	-0.107	0.240	6.57E-01	0.081	-0.344	0.181	6.02E-02
rs4665852	2	26570587	A	G	3.00	0.154	---	0.321	-0.237	0.067	4.38E-04	0.744	-0.119	0.170	4.88E-01	0.466	-0.195	0.097	4.69E-02

Chr: chromosome; Freq: frequence of effect allele. SE: standard error.

Log_{10} BF: log_{10} Bayes' factor in favor of association. $P_{posterior}$: posterior probability of heterogeneity.

Direction: effect directions (coded as ? for missing, + for positive allelic effect for effect allele A1, and - for negative allelic effect for effect allele A1)

REFERENCE

1 Ministry of Health PsRoC, UNAIDS, World Health Organization. 2011 Report on the estimation of HIV/AIDS epidemic in China. *Beijing* (2012). Available at: http://www.chinaaids.cn/fzdt/zxdd/201201/t20120129_1745902.htm (Date of access: 15th April 2015)

2 Yaping, Q., zhengtao, C. & Candong, W. Investigation of polymorphism of DYS287 from five ethincs in Yunnan province of China. *CHINESE JOURNAL OF MEDICAL GENETICS* **16** (1999).

3 Patterson, N., Price, A. L. & Reich, D. Population Structure and Eigenanalysis. *PLoS Genet* **2**, e190 (2006).

4 Price, A. L. *et al.* Principal components analysis corrects for stratification in genome-wide association studies. *Nat Genet* **38**, 904-909 (2006).

5 Consortium, T. I. H. The International HapMap Project. *Nature* **426**, 789-796 (2003).

6 Pruim, R. J. *et al.* LocusZoom: regional visualization of genome-wide association scan results. *Bioinformatics* **26**, 2336-2337 (2010).

7 Morris, A. P. Transethnic Meta-Analysis of Genomewide Association Studies. *Genet Epidemiol.* **35**, 809-822 (2011).
